# Simplified three-dimensional tissue clearing and incorporation of colorimetric phenotyping

**DOI:** 10.1038/srep30736

**Published:** 2016-08-08

**Authors:** Kevin Sung, Yichen Ding, Jianguo Ma, Harrison Chen, Vincent Huang, Michelle Cheng, Cindy F. Yang, Jocelyn T. Kim, Daniel Eguchi, Dino Di Carlo, Tzung K. Hsiai, Atsushi Nakano, Rajan P. Kulkarni

**Affiliations:** 1 Department of Bioengineering, University of California, Los Angeles, CA 90095, Los Angeles, USA; 2 Division of Cardiology, Department of Medicine, David Geffen School of Medicine at UCLA, CA 90095, Los Angeles, USA; 3 Department of Molecular, Cellular, and Developmental Biology, University of California, Los Angeles, CA 90095, Los Angeles, USA; 4 Division of Dermatology, Department of Medicine, David Geffen School of Medicine at UCLA, CA 90095, Los Angeles, USA; 5 Department of Neurobiology, David Geffen School of Medicine at UCLA, CA 90095, Los Angeles, USA; 6 Division of Infectious Diseases, Department of Medicine, David Geffen School of Medicine at UCLA, CA 90095, Los Angeles, USA; 7 California NanoSystems Institute, UCLA, CA 90095, Los Angeles, USA; 8 Jonsson Comprehensive Cancer Center, UCLA, CA 90095, Los Angeles, USA; 9 Eli and Edythe Broad Center of Regenerative Medicine and Stem Cell Research at UCLA, CA 90095, Los Angeles, USA.

## Abstract

Tissue clearing methods promise to provide exquisite three-dimensional imaging information; however, there is a need for simplified methods for lower resource settings and for non-fluorescence based phenotyping to enable light microscopic imaging modalities. Here we describe the simplified CLARITY method (SCM) for tissue clearing that preserves epitopes of interest. We imaged the resulting tissues using light sheet microscopy to generate rapid 3D reconstructions of entire tissues and organs. In addition, to enable clearing and 3D tissue imaging with light microscopy methods, we developed a colorimetric, non-fluorescent method for specifically labeling cleared tissues based on horseradish peroxidase conversion of diaminobenzidine to a colored insoluble product. The methods we describe here are portable and can be accomplished at low cost, and can allow light microscopic imaging of cleared tissues, thus enabling tissue clearing and imaging in a wide variety of settings.

The ability to phenotype tissues in three dimensions is important for both clinical and research settings; the development of tissue clearing methods allows phenotyping of intact tissues with high resolution^1^. These methods either involve stabilization of proteins and nucleic acids coupled with removal of light-scattering lipids or the use of chemicals to remove lipids and suppress refractive index mismatches^2^,^3^,^4^,^5^. However, despite these advances, most methods involve complex steps and expensive or hazardous chemicals. Thus, a tissue clearing method is needed that is simple, inexpensive, easy-to-follow and truly portable, free of bulky or expensive equipment or reagents. The CLARITY protocol has comparatively few steps and utilizes relatively inexpensive reagents, but its initial implementation required an electrical setup for electrophoresis of detergent solution through the tissue-hydrogel hybrid, which limits its portability^6^. The passive CLARITY technique (PACT) removes this step and utilizes passive diffusion for clearing, but still requires a low-oxygen tension environment generated through vacuum pumping and nitrogen gas backfilling^4^,^5^.

A second limitation of most clearing methods is the use of fluorescently labeled molecules and probes to generate specific labeling and contrast^7^. Many laboratories in low-resource settings do not have access to fluorescence microscopy. In addition several portable microscopy setups (such as those utilizing holographic microscopy or cell phone cameras) cannot image fluorescent labels^8^,^9^. Finally, in certain cases, phase contrast or other light microscopic methods can generate additional information beyond what is available through fluorescence microscopy. Thus, there is a need for colorimetric methods that do not rely on the use of fluorescence to generate contrast for analyzing cleared tissues with light microscopic methods. These colorimetric methods would either employ stains or chemical processes that caused a visible color change to occur at specific sites of interest within the tissue and could be visualized with visible white light (such as from a halogen lamp).

Here we describe a more portable implementation of tissue clearing, based on PACT, termed SCM (simplified CLARITY method). We show that polymerization and clearing can be performed with minimal equipment. Furthermore, we show that we can utilize colorimetric methods for specific labeling and imaging of tissues based on the diaminobenzidine reaction and the resulting tissues can be imaged with visible light microscopy techniques. These methods hold promise to enable tissue processing, clearing, and imaging at reduced cost and to enable additional, complementary microscopic methods to be utilized to image the resulting tissues.

## Results

**Figure 1:**
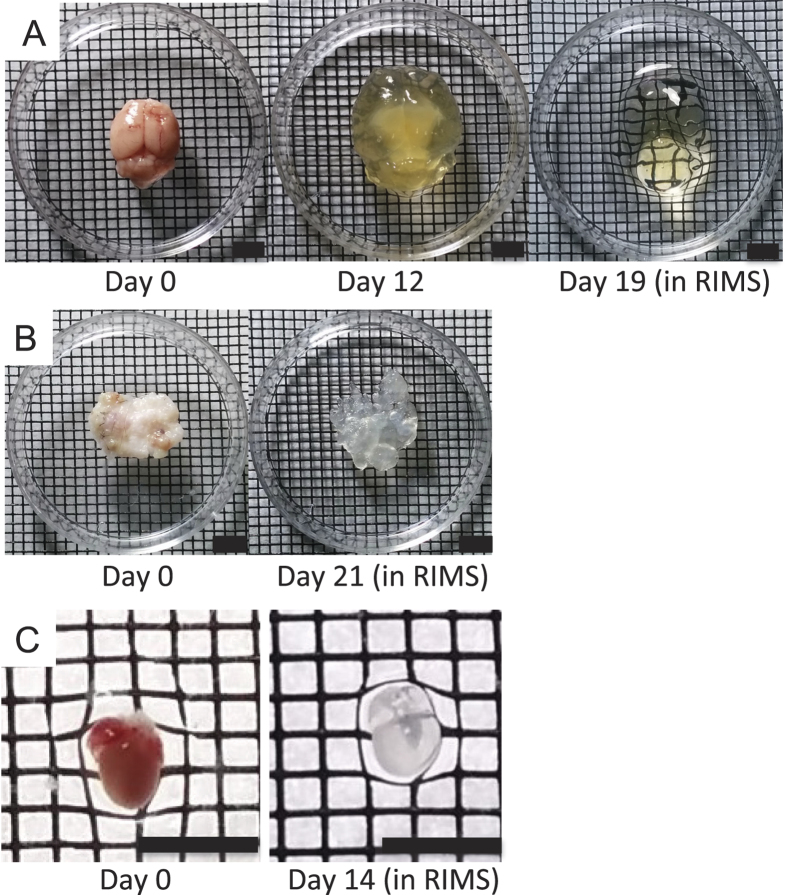
SCM is effective for clearing a diverse variety of tissues. Photomicrographs of different tissues processed with the simplified CLARITY method (SCM). (**A**) Adult mouse brain before SCM (left), at day 12 of clearing (middle) and after clearing was complete and sample placed in RIMS (right). (**B**) Human basal cell carcinoma (BCC) before (left) and after clearing (right). (**C**) Neonatal mouse heart (P1) before (left) and after clearing (right). Scale bars = 4.2 mm.

The simplified CLARITY method (SCM) generates tissue-hydrogel hybrids with acrylamide by increasing the concentration of the initiator for the polymerization reaction, thus eliminating the need for vacuum purging and nitrogen backfilling. Solutions of initiator must be freshly made as we have found that polymerization efficiency decreased with increasing age of the initiator, likely due to oxidation. Figure 1 depicts macroscopic photographs of a mouse brain before, during, and after clearing and a human basal cell carcinoma and mouse heart before and after clearing, all processed using SCM. The overall clearing time is directly dependent on the thickness of the tissue, clearing temperature, as well as the extent of cross-linking prior to polymerization. For example, a 0.5-mm section of mouse cardiac tissue can be cleared in several hours, while an adult mouse brain (which is several centimeters in thickness) can be cleared at 37 °C in approximately two to three weeks. If available, the use of a rotating water bath and an increase in temperature to 42 °C can reduce the clearing time. SCM clearing times are similar to those of PACT CLARITY.

It is critical to ensure complete washing of samples with multiple phosphate buffered saline rinses (or other buffered aqueous solutions) prior to cross-linking with paraformaldehyde, in order to thoroughly remove red blood cells before they lyse. Red blood cell accumulation and lysis in samples may lead to hemoglobin polymerization, which contributes to autofluorescence. Although a method to decolorize heme using aminoalcohol has been described^2^,^3^, we have found that this chemical treatment reduced YFP fluorescence when utilized to remove hemoglobin from cardiac tissue slices (Supplementary Figure 1). Thus, we sought to develop a method that employed less harsh chemicals and did not reduce endogenous fluorescence.

**Figure 2:**
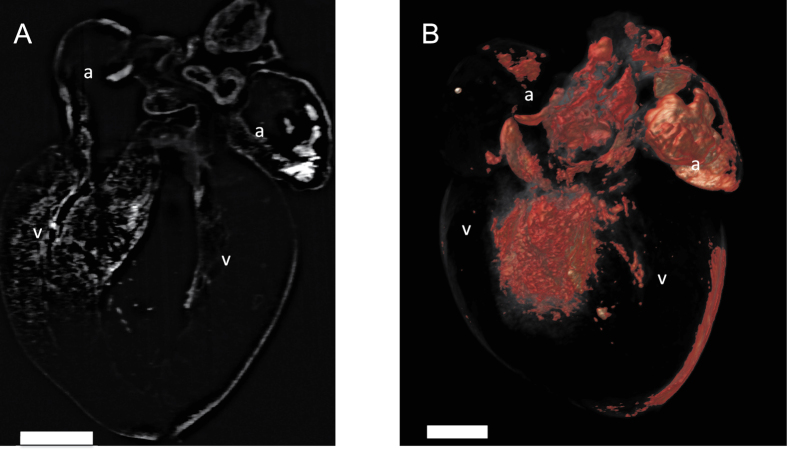
Light sheet imaging of SCM cleared hearts enables high-resolution structural visualization. (**A**) A coronal section of P1 mouse heart (R26R-YFP crossed with Sln-Cre) demonstrating YFP fluorescence in the atria and part of the ventricular septum. Structural features can also be seen from tissue autofluorescence. (**B**) A three-dimensional rendering of the entire volume of the P1 mouse heart from A. YFP fluorescence is shown in yellow, (and tissue autofluorescence is shown in red). Trabecular networks and structural features can be visualized with light sheet microscopy. a = atrium, v = ventricle. Scale bars = 0.5 mm.

The SCM-generated tissues preserve endogenous genetic fluorescence (if present) and are compatible with most immunohistochemical techniques including labeling with specific antibodies. For fluorescently labeled specimens, we utilized light sheet microscopy (LSM) to image fluorescent labels within intact cleared tissues, to enable the deepest tissue imaging possible at a fast rate, as we have previously shown that LSM offers the greatest depth of view and most rapid image acquisition as compared to commercially available confocal microscopy systems^10^. Light sheet microscopy has been described as being optimal for CLARITY-cleared tissues as it maximizes imaging depth and enables fast large-volume visualization^11^,^12^. Figure 2 depicts images of cleared neonatal mouse hearts with a sarcolipin-Cre (Sln-Cre; R26R-YFP) genotype^13^,^14^. These mouse hearts exhibit yellow fluorescent protein (YFP) genetic labelling and were imaged using a custom built light-sheet microscope setup (Fig. 2). YFP fluorescence is observed in the atria and ventricular septa. In addition, these tissues can be imaged with confocal microscopy or commercially available light sheet microscopy devices. If neither confocal nor light sheet microscopy is available, then thinner sections of SCM cleared and fluorescently-labeled tissues can be imaged with epifluorescence microscopy. Recent research demonstrates that mouse brain can be imaged to a depth of several hundred microns using epifluorescence microscopy^15^.

Almost all tissue clearing methods have been developed to enable fluorescence imaging. While useful in many cases, this excludes the use of light microscopic techniques such as phase microscopy which can provide complementary information about tissue samples. In addition, many of the portable microscopy solutions such as those utilizing holographic lens-free microscopy or cell phone cameras with additional attachments, are based on light microscopy. To allow cleared tissues to be imaged with additional non-fluorescence based modalities, we thus wished to determine parameters to enable color-based contrast imaging using colorimetric labeling of SCM processed tissues. As a proof of concept, we labeled parvalbumin neurons in slices of adult mouse cortex. The parvalbumin neurons are a unique subset of neurons that are few in number and help to synchronize cortical signals; they can be labeled specifically using anti-parvalbumin primary antibodies. Instead of utilizing a fluorescently-labeled secondary antibody, we utilized secondary antibodies labeled with horseradish peroxidase. On exposure to the substrate 3,3′-diaminobenzidine tetrahydrochloride (DAB), the horseradish peroxidase converts the DAB to an insoluble brown product of micron-sized particles that can be visualized with epi-illumination visible light microscopy or phase microscopy^16^. Care is needed when using HRP and DAB, as exposure for too long will cause non-specific staining and potential diffusion of the insoluble product from the site of reaction, whereas insufficient exposure time or concentration will yield no visible reaction. Of note, sodium azide should not be added to the buffers prior to adding DAB, as it is known to inactivate horseradish peroxidase^17^. Once the reaction is completed, sodium azide may be added to prevent microbial growth.

**Figure 3:**
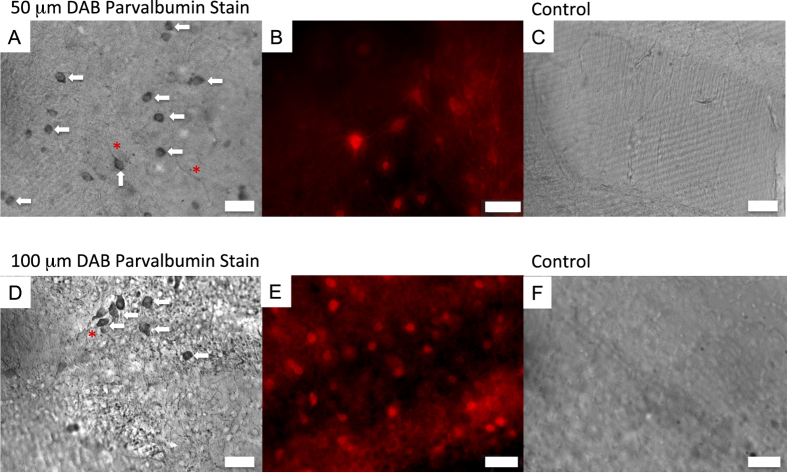
Parvalbumin neurons can be localized in brain sections using DAB colorimetric staining. (**A**) 50 μm sections of cleared adult mouse brain labeled with anti-parvalbumin primary and horseradish peroxidase-labeled secondary antibodies, followed by incubation with DAB substrate. The neuronal cell bodies stain with brown color, cell bodies are labeled with arrows and axonal processes are labeled with asterisks. (**B**) 50 μm section of cleared adult mouse brain labeled with parvalbumin primary and Alexa 594-labeled secondary antibody. Cell bodies and neuronal processes are visualized; the axonal processes appear indistinct due to out-of-focus fluorescence from the thick section. (**C**) Control 50 μm section labeled with only HRP-labeled secondary antibody followed by DAB substrate (no primary antibody). As expected, no neurons retain label. (**D**) 100 μm sections of cleared adult mouse brain labeled with anti-parvalbumin primary and HRP-labeled secondary antibodies, followed by incubation with DAB substrate. Cell bodies and axonal processes retain brown-colored DAB label; selected cell bodies are labeled with arrows and identifiable axonal processes are labeled with arrowheads. (**E**) 100 μm sections of cleared adult mouse brain labeled with anti-parvalbumin primary and Alexa 594-labeled secondary antibody. (**F**) Control 100 μm section labeled as described in C. Scale bars = 50 μm.

To determine the correct parameters to enable DAB staining, we utilized 50 and 100 μm sections of adult mouse brain tissues. We determined that for these 50 μm and 100 μm sections, the secondary-labeled tissue should be incubated with DAB for 2–6 minutes for maximum tissue contrast and minimal background staining, at the concentration of DAB solution we utilized. Longer DAB incubation times may cause non-specific staining or diffusion of the particles away from the reaction site. The exact incubation time depends on the thickness of the tissue and the concentration of antibodies utilized. We then imaged the resulting DAB-stained tissues using epi-illumination light microscopy and determined that we could specifically visualize parvalbumin neuronal bodies and some of the dendritic and axonal processes. Figure 3 shows the resulting images (Fig. 3A,D), compared with similar sections of mouse brain labeled with fluorescently labeled secondary antibody imaged with epifluorescence microscopy (Fig. 3B,E). Note that the cell bodies (arrows) and some axonal processes (asterisks) can be labeled with DAB stain (Fig. 3A,D), though the strength of signal depends on the incubation time with DAB.

## Discussion

Tissue clearing offers an unprecedented ability to image critical structures and features in three dimensions and offers a complementary approach to traditional histopathological analysis using formalin-fixed and paraffin-embedded tissue sections. The methods we have described are a significant improvement and simplification over currently available techniques and will allow for tissue clearing to be utilized in almost any setting due to its independence from bulky processing equipment. The main differences of SCM to previously published protocols involve the reduction in clearing reagent use and the simplification of the protocol to eliminate the need for reducing oxygen tension in the sample and thus the need for vacuum pumps or nitrogen gas sources while maintaining sample fidelity and integrity and comparable clearing times. The resulting cleared tissues maintain endogenous expression of genetically-encoded fluorescent proteins, which are compatible with a broad variety of antibody-labeling approaches, and most importantly now have the ability to be imaged with light microscopy using the colorimetric DAB stain approach as first reported here. The resulting images appear similar to light microscopic histological images, but also allow intact deeper-tissue imaging. The use of colorimetric stains allows tissues to be analyzed using light microscopy and phase contrast microscopy and potentially with portable microscopic approaches. We did not observe appreciable diffusion of the DAB particles in the tissue, thus demonstrating the stain specifically localizes to and labels features of interest. In addition, other color-based contrast methods can also be incorporated to allow multiplexed colorimetric phenotyping with high resolution.

Tissues cleared with SCM can be visualized with a variety of microscopic systems. Tissues with genetic fluorescent proteins or labeled with fluorescent markers can be imaged with several modalities, including epifluorescence, confocal, or light sheet microscopy. We have primarily utilized light sheet microscopy to enable rapid imaging of thick tissues; however, confocal or epifluorescence microscopy can also yield structural and phenotypic information in an appropriately labeled sample^15^. SCM-cleared tissues labeled colorimetrically can be imaged with epi-illumination light microscopy and phase contrast microscopy, which we have demonstrated here, and potentially with other non-fluorescence based modalities, including reflectance confocal microscopy and lens-free holographic microscopy^18^,^19^.

SCM and the colorimetric contrast-generating methods can allow tissue clearing approaches to have a wider reach. The ability to utilize phase contrast and other non-fluorescence based microscopic approaches can increase the information that can be gained from such tissues. We envision that these methods can also be utilized in research laboratories in lower resource settings, as they are simple to perform and do not require complex or costly equipment.

## Materials and Methods

### Preparation of tissues

Mouse tissues were harvested according to the UCLA IACUC guidelines and all experimental protocols were approved by the IACUC. Briefly, mice were deeply anesthetized with pentobarbital and perfused transcardially with saline followed by 4% paraformaldehyde in phosphate-buffered saline (PBS). Human skin cancer tissue samples were obtained from patients undergoing excision of their cancers after appropriate informed consent and under approval of UCLA IRB#12–01195, in accordance with UCLA IRB guidelines. Tissue samples were obtained from sections of tumors not necessary for diagnostic or margin control purposes and varied in size depending on the size of the original skin cancer. All tissues were rinsed in PBS three times for ten minutes and then placed in 4% paraformaldehyde (Electron Microscopy Sciences) at 4 °C overnight. Brain tissues to be sectioned were then embedded in 3% Bactoagar and free-floating coronal sections (50, 100 μm) were collected using a vibratome (Leica Microsystems).

The tissues were then placed in a 4% acrylamide solution (Bio-Rad) along with 0.5% w/v of the photoinitiator 2,2′-Azobis[2-(2-imidazolin-2-yl)propane]dihydrochloride (VA-044, Wako Chemicals USA); this is double the concentration of the original PACT CLARITY protocol. The tissues were then incubated overnight at 4 °C followed by incubation at 37 °C for 2–3 hours to initiate polymerization of the acrylamide. After polymerization, the tissues were rinsed with PBS and then placed into a clearing solution comprised of 8% w/v sodium dodecyl sulfate (SDS, Sigma Aldrich) and 1.25% w/v boric acid (Fisher) (pH 8.5). The samples were incubated at 37 °C until cleared. Tissues were then rinsed for one day in 1X PBS after clearing to remove residual SDS.

### Labeling of mouse brain tissues

To prepare cleared mouse brain tissues for the DAB stain of parvalbumin-containing neurons (Pab), rat monoclonal anti-mouse Pab primary antibodies (ImmunoStar) were diluted in a 1:200 volume/volume ratio with blocking solution, which was comprised of 1% v/v goat serum and 0.1% v/v Tween-20 in PBS (hereafter referred to as goat blocking solution).

The cleared mouse brain sections were incubated in 200 μL of the primary antibody-blocking solution mixture for 48 hours shaking at room temperature. Subsequently, the cleared brain sections were washed with 1x PBS for 24 hours with rotation at room temperature. For fluorescent labeling, goat monoclonal anti-rat antibodies conjugated with Alexa-594 (Pierce Antibodies) were diluted in a 1:200 v/v ratio with goat blocking solution to form the secondary antibody-blocking solution mixture. For colorimetric imaging, goat monoclonal anti-rat antibodies conjugated with horseradish peroxidase enzyme (Thermo Scientific Pierce Antibodies) were diluted in a 1:200 volume/volume ratio with goat blocking solution. The cleared mouse brain sections were incubated with the appropriate secondary antibody-blocking solution mixture for 48 hours with rotation at room temperature. Afterwards, the cleared mouse brain sections were washed with 1x PBS for 24 hours with rotation at room temperature.

To prepare the DAB solution for colorimetric imaging, a 3,3′-Diaminobenzidine (DAB) solution was formulated with 250 μL of 1% w/v 3,3′-Diaminobenzidine (Sigma), 250 μL of 0.3% hydrogen peroxide solution (Sigma), and 4.5 mL of DI water. The solution was then adjusted to a pH of 7.4 with 1N sodium hydroxide (Fisher Chemical).

The cleared mouse brain sections labeled with the horseradish peroxidase secondary antibody were placed in the DAB solution for 2 minutes, 4 minutes, and 6 minutes respectively to form light, medium, and dark stains. These samples were then washed with 1x PBS to remove the excess DAB solution. The DAB stained samples were placed in refractive index matching solution (RIMS) for imaging and storage. The RIMS formulation is as follows: to make 30 mL RIMS, dissolve 40 grams of Histodenz (Sigma) in 0.02 M Phosphate buffer (Sigma) with 0.05% w/v sodium azide (Sigma) and syringe filtered through a 0.2 μm filter. The solution can be stored at room temperature.

### Imaging of tissues

#### Light sheet imaging

Light sheet imaging was carried out on a home-made system (Supplementary Figure 2). A diode-pumped solid-state laser (LMM-GB1, Laserglow Technologies, Toronto, Canada) was used as the 532 nm illumination source. The initial diameter of laser beam was 2 mm, and then light passed through a 5x achromatic beam expander (GBE05-A, Thorlabs Inc, New Jersey, USA). A beam splitter (BS013, Thorlabs Inc, New Jersey, USA) was employed to form dual-illumination onto the sample from opposite directions. Each beam was focused by a plano-convex cylindrical lens (*f* = 50 mm, LJ1695RM-A, Thorlabs Inc, New Jersey, USA) and was then reshaped by a group of achromatic doublets (AC254-060-A, AC254-100-A, Thorlabs, New Jersey, USA). After passing through an *f* = 150 mm lens, the beam expanded to a sheet with the width of 40 mm, as well as the waist of 17 μm. The detection module was installed perpendicular to the illumination plane, and it was composed of a stereo microscope (MVX10, Olympus, Japan) with a 1x magnification objective (Numerical aperture, NA: 0.25), a scientific CMOS (ORCA-Flash4.0LT, Hamamatsu, Japan) and optical filters (Semrock, New York, USA). The sample with RIMS and 1% agarose solution was embedded in a borosilicate glass tubing with an inner diameter of less than 6 mm, and then the mounted sample was placed on a motorized translational stage. Both the sample and its holder, but not the translational stage, were immersed in a transparent chamber filled with 99.5% glycerol to match the refractive index among different materials. Illumination and detection were controlled by a computer with dedicated SSD RAID 0 storage for fast data streaming.

#### Epi-illumination and epifluorescence microscopy

Colorimetrically labeled tissues were imaged using a Zeiss Axiovert inverted microscope with the included epi-illumination light source. Brain sections labeled with Alexa 594-labeled secondary antibodies were imaged using this Zeiss Axiovert microscope with a xenon-mercury arc lamp and appropriate excitation and emission filters. Images were collected using a Photometrics CoolSnap HQ2 camera.

## Additional Information

**How to cite this article**: Sung, K. *et al.* Simplified three-dimensional tissue clearing and incorporation of colorimetric phenotyping. *Sci. Rep.* **6**, 30736; doi: 10.1038/srep30736 (2016).

## Supplementary Material

Supplementary Information
