# Supplementary material for: Simplified three-dimensional tissue clearing and incorporation of colorimetric phenotyping
Source: Sci Rep. 2016 Aug 8;6:30736. doi: 10.1038/srep30736 (PMC4976371; doi:10.1038/srep30736)

Supplementary Information

Simplified three-dimensional tissue clearing and incorporation of colorimetric phenotyping

Kevin Sung,[1] Yichen Ding[2], Jianguo Ma[2], Harrison Chen[1], Vincent Huang[3], Michelle Cheng[4], Cindy F. Yang[5], Jocelyn T. Kim[6], Daniel Eguchi[4], Dino Di Carlo[1,7,8], Tzung K. Hsiai[2,7], Atsushi Nakano[3,8,9], Rajan P. Kulkarni[1,4,7,8,*]

Supplementary Figure 1: YFP fluorescence signal is lost from cardiac tissues on incubation with aminoalcohol. 100 um sections of mouse cardiac tissue expressing YFP were incubated in aminoalcohol solution (25% weight% *N,N,N',N'*-tetrakis(2-hydroxypropyl)ethylenediamine in PBS) for 9 hours and fluorescence readings taken with a plate reader at 0, 3, and 9 hours. As shown in the graph, there is significant loss of native YFP signal from the tissue at 9 hours in the sample incubated with aminoalcohol compared to the control sample incubated in PBS alone.

Supplementary Figure 2: Schematic illustration of light sheet imaging system. CL: cylindrical lens; TL: tube lens; M: mirror.

Supplementary Figure 1

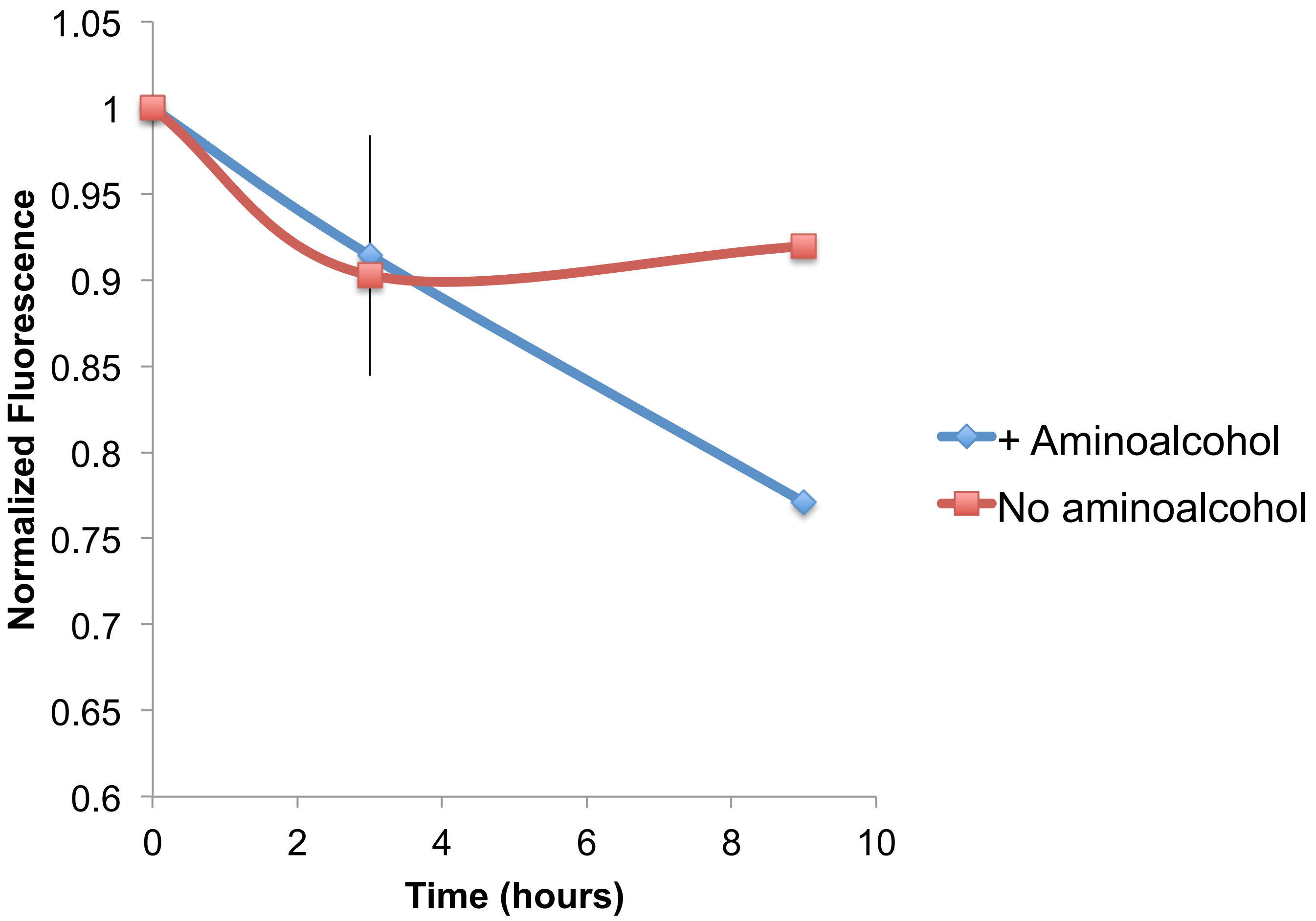

Supplementary Figure 2

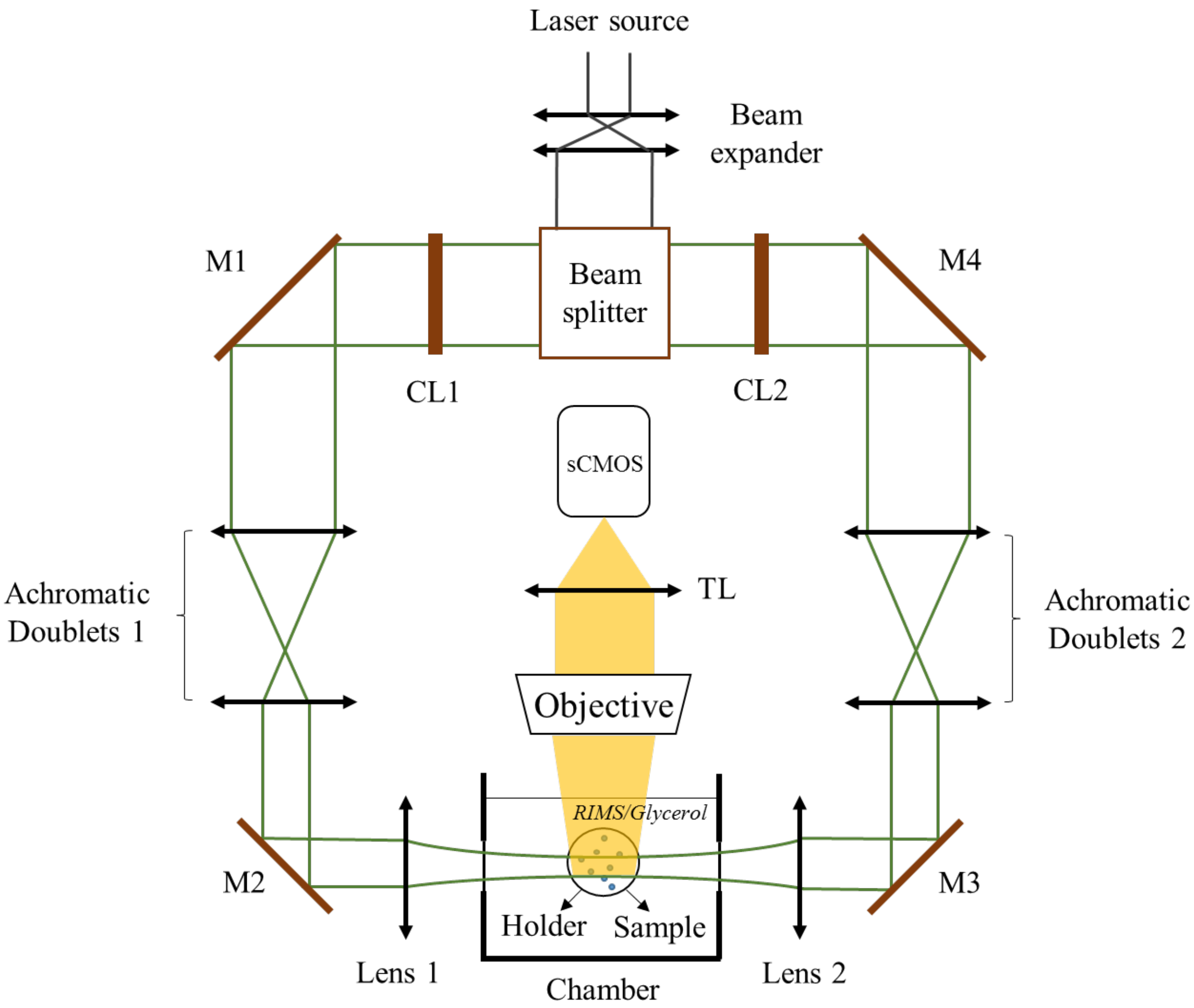

Supplement: Supplementary Information [file srep30736-s1.pdf]
